# Insights into phosphatidic acid phosphatase and its potential role as a therapeutic target

**DOI:** 10.1016/j.jbior.2025.101074

**Published:** 2025-01-03

**Authors:** George M. Carman, Geordan J. Stukey, Ruta Jog, Gil-Soo Han

**Affiliations:** Department of Food Science and the Rutgers Center for Lipid Research, Rutgers University, New Brunswick, NJ, 08901, USA

**Keywords:** Phospholipid, Triacylglycerol, Phosphatidic acid, Diacylglycerol, Nem1-Spo7 protein phosphatase, Protein kinase, Sertraline, Propranolol, Yeast, *Saccharomyces cerevisiae*

## Abstract

Phosphatidic acid phosphatase, a conserved eukaryotic enzyme that catalyzes the Mg^2+^-dependent dephosphorylation of phosphatidic acid to produce diacylglycerol, has emerged as a vital regulator of lipid homeostasis. By controlling the balance of phosphatidic acid and diacylglycerol, the enzyme governs the use of the lipids for synthesis of the storage lipid triacylglycerol and the membrane phospholipids needed for cell growth. The mutational, biochemical, and cellular analyses of yeast phosphatidic acid phosphatase have provided insights into the structural determinants of enzyme function with the understanding of its regulation by phosphorylation and dephosphorylation. The key role that the enzyme plays in triacylglycerol synthesis indicates it may be a potential drug target to ameliorate obesity in humans. The enzyme activity, which is critical to the growth and virulence of pathogenic fungi, is a proposed target for therapeutic development to ameliorate fungal infections.

## 1. PAP plays a key role in lipid homeostasis

**Fig. 1.**
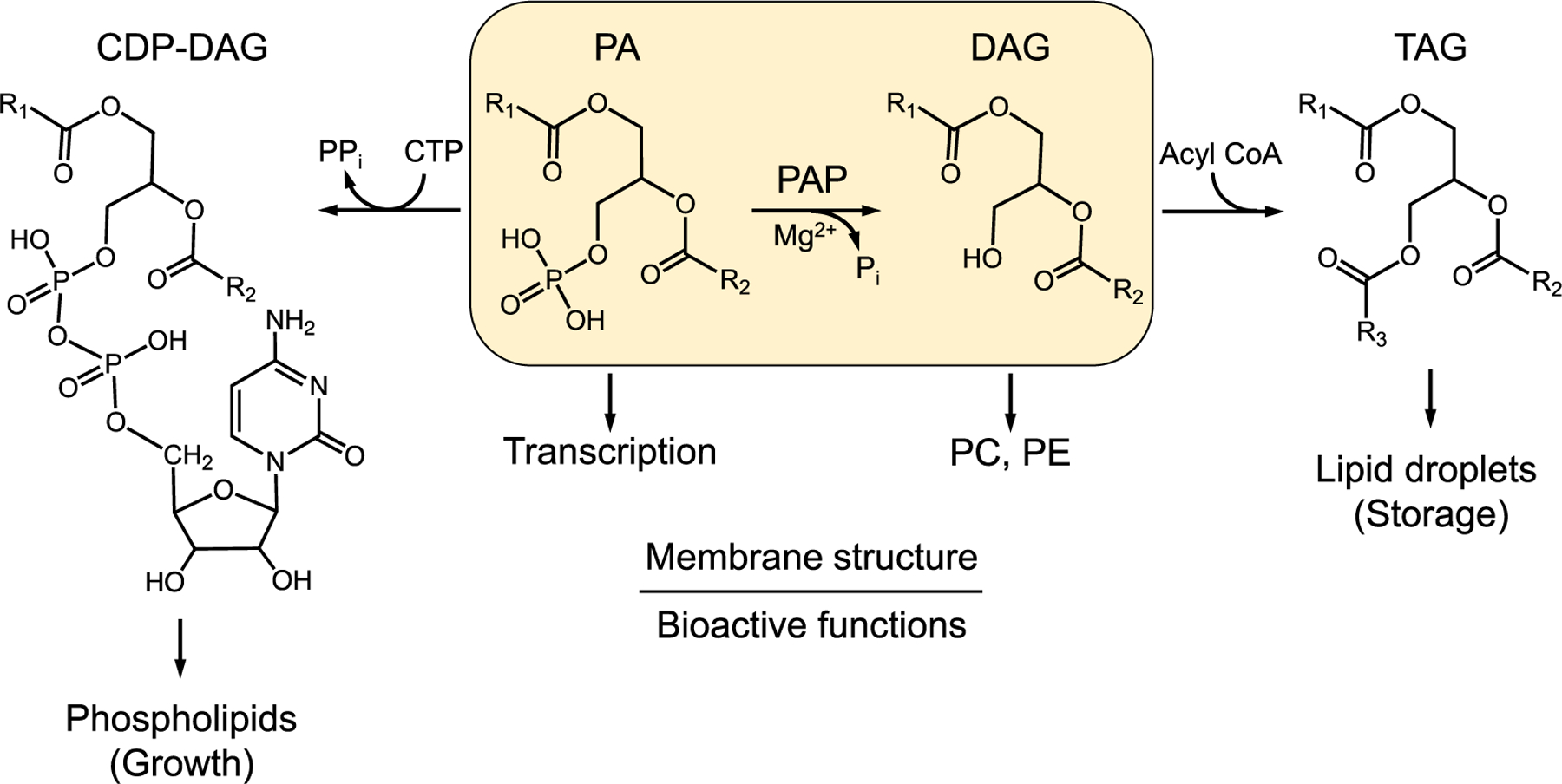
Role of PAP in lipid synthesis and cell physiology. The structures of CDP-DAG, PA, DAG, and TAG are shown. PAP catalyzes the Mg^2+^-dependent dephosphorylation of PA to form DAG. PAP plays a key role in the use of PA for the CDP-DAG-dependent synthesis of membrane phospholipids needed for cell growth or the synthesis of the storage lipid TAG via DAG. The DAG produced by the PAP reaction may also be used for the synthesis PC and PE when cells are supplemented with choline and ethanolamine, respectively. Additional roles of PA and DAG in cell physiology are indicated. More comprehensive pathways of lipid synthesis may be found in Refs. (Carman and Han, 2011; Henry et al., 2012).

Lipids are molecules that perform a variety of essential functions in eukaryotic organisms. They are responsible for storing energy in the form of fat and for the bilayer of membranes that compartmentalize cellular processes. Lipids also help with the absorption of fatsoluble vitamins and synthesis of steroid hormones. Having too much or too little of some lipids may lead to a variety of lipid-based diseases. One enzyme that has emerged as a vital regulator of lipid homeostasis in eukaryotes is phosphatidic acid (PA) phosphatase (PAP)^1,2^ (Donkor et al., 2007; Eastmond et al., 2010; Golden et al., 2009; Han et al., 2006; Han and Carman, 2010; Nakamura et al., 2009; Péterfy et al., 2001; Ugrankar et al., 2011; Valente et al., 2010), which catalyzes the Mg^2+^-dependent dephosphorylation of PA to produce diacylglycerol (DAG) (Kates, 1955; Smith et al., 1957) (Fig. 1). PAP plays a major role in lipid homeostasis by controlling the cellular levels of its substrate PA and product DAG, both of which are key intermediates for the synthesis of the storage lipid TAG and the membrane phospholipids needed for cell growth (Carman and Han, 2019; Kwiatek et al., 2020; Reue and Brindley, 2008; Reue and Wang, 2019; Vance, 2004) (Fig. 1). PA may be converted into CDP-DAG, which is then utilized for the synthesis of major phospholipids that include phosphatidylserine, phosphatidylethanolamine, phosphatidylcholine, phosphatidylinositol, phosphatidylglycerol, and cardiolipin (Carman and Han, 2011; Henry et al., 2012; Kwiatek et al., 2020; Reue and Brindley, 2008; Vance, 2004) (Fig. 1). The DAG produced by the PAP reaction is channeled into TAG for storage, and under certain conditions (i.e., choline and ethanolamine supplementation, respectively, to auxotrophic mutants defective in CDP-DAG-dependent phospholipid synthesis) into phosphatidylcholine and phosphatidylethanolamine (Chae et al., 2012; Han et al., 2006; Kwiatek et al., 2020; Reue and Brindley, 2008; Vance, 2004) (Fig. 1).

PA and DAG also function in lipid signaling pathways (Dey et al., 2017; Exton, 1990, 1994; Kudo et al., 2020; Kwiatek et al., 2020; Testerink and Munnik, 2005; Waggoner et al., 1999), vesicular trafficking (Baron and Malhotra, 2002; Lehel et al., 1995; Maissel et al., 2006; Morris, 2007; Roth, 2008), membrane fission/fusion events (Blackwood et al., 1997; Chernomordik et al., 1995; Goni and Alonso, 1999; Koter et al., 1978; Liao and Prestegard, 1979; Weigert et al., 1999), and expression of phospholipid synthesis genes (Carman and Henry, 2007; Gaspar et al., 2022; Han and Carman, 2017) (Fig. 1). The importance of maintaining the PA-DAG balance by the PAP enzyme to lipid homeostasis is highlighted in yeast, mice, and humans by a host of cellular defects (e.g., aberrant nuclear membrane morphology, defects in lipid droplet formation, fatty acid-induced lipotoxicity, defects in vacuole fusion and autophagy, apoptosis, and reduced chronological life span) and lipid-based diseases (e.g., lipodystrophy, obesity, inflammation, insulin resistance, peripheral neuropathy, type 2 diabetes) that are associated with the loss or overexpression of the PAP enzyme (Carman and Han, 2019; Csaki et al., 2013; Kwiatek et al., 2020; Pascual and Carman, 2013; Reue, 2009; Reue and Brindley, 2008; Reue and Donkor, 2007; Reue and Dwyer, 2009; Santos-Rosa et al., 2005; Xu et al., 2006; Zhang et al., 2014; Zhang and Reue, 2017).

## 2. PAP is conserved from yeast to humans

Much knowledge of PAP and its roles in lipid metabolism has been derived from studies using the simple eukaryotic model organism *Saccharomyces cerevisiae* (Carman and Han, 2009, 2011, 2019; Henry et al., 2012; Kwiatek et al., 2020; Pascual and Carman, 2013). Studies with *S. cerevisiae* have been pivotal in identifying the gene-enzyme relationship of mammalian PAP. While many attempts had been made to purify PAP from diverse organisms, the enzyme was purified to near homogeneity only from *S. cerevisiae* (Lin and Carman, 1989), and its sequence information led to the discovery of *PAH1* as the gene encoding the enzyme (Han et al., 2006). The human *LPIN1*-PAP relationship was discovered (Han et al., 2006) using the sequence information that shows the conservation of the N-LIP and HAD-like domains (catalytic core components) in the yeast and human enzymes (Péterfy et al., 2001). *S. cerevisiae* Pah1 has been extensively examined for its regulation, and what has been learned from the yeast enzyme has directly led to discoveries with mammalian PAP enzymes. While some differences exist in their protein architectures (Gu et al., 2021; Karanasios et al., 2013; Stukey et al., 2023), the yeast and human PAPs show conservation in the basic mechanism of enzyme action through its catalytic core and in the enzyme regulation by phosphorylation and dephosphorylation (Carman and Han, 2006, 2009; Stukey et al., 2024b).

## 3. Phosphorylation and dephosphorylation of Pah1 regulates its location, function, and stability

**Fig. 2.**
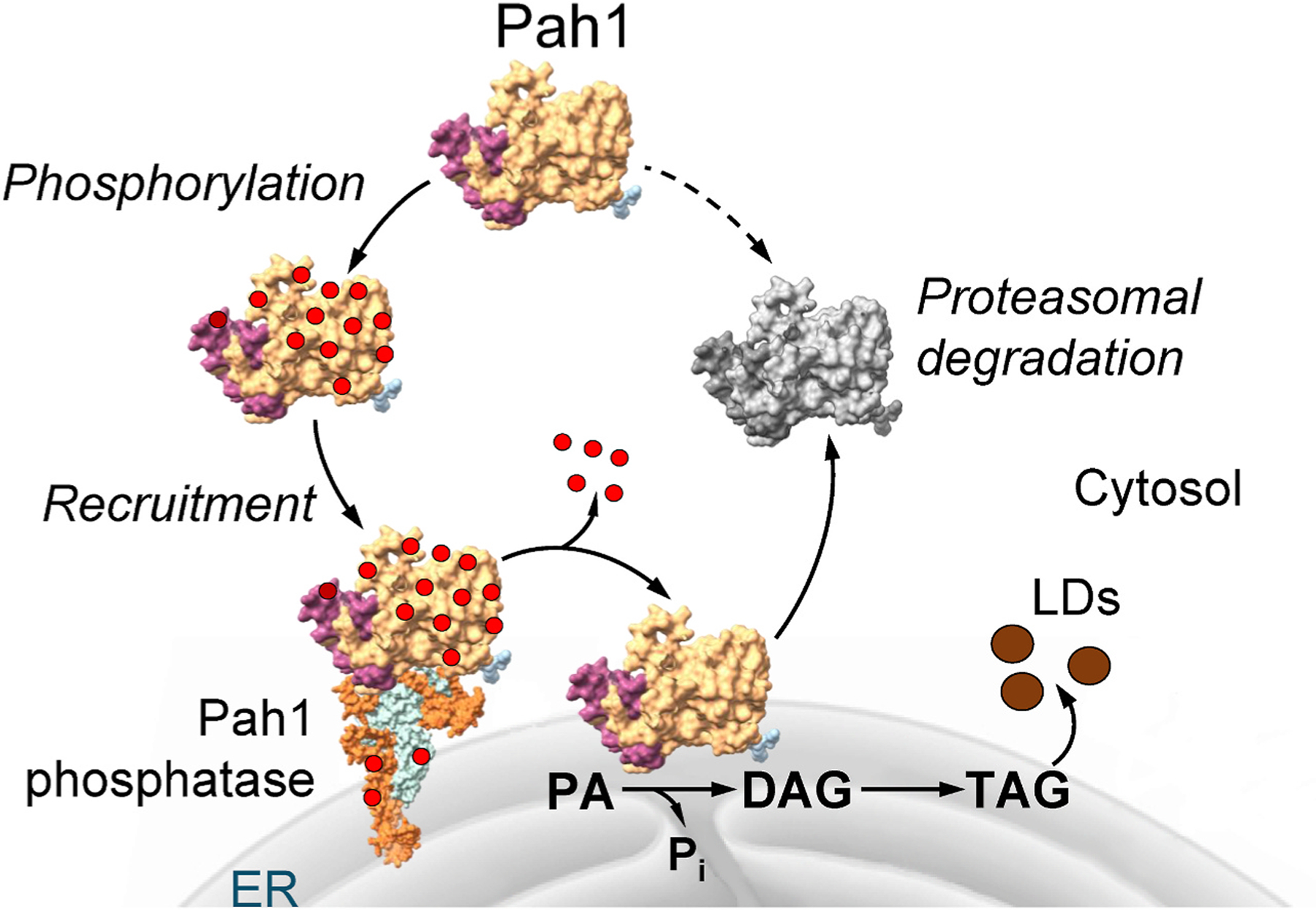
Phosphorylation and dephosphorylation of Pah1 regulates its location, function, and stability. Following its expression, Pah1 in the cytosol is unstable and highly phosphorylated for protection against proteasomal degradation. Phosphorylated Pah1 is stable, but functionally inactive due to its sequestration in the cytosol apart from its membrane-associated substrate. The Pah1 phosphatase, which is composed of Nem1 and Spo7, recruits and dephosphorylates Pah1 at the nuclear/ER membrane. Following its dephosphorylation, Pah1 associates with the membrane and catalyzes the dephosphorylation of PA to produce DAG, which is then acylated to TAG that is stored in lipid droplets (LDs). Following rounds of catalysis, Pah1 dissociating from the nuclear/ER membrane is subject to proteasomal degradation (indicated by *gray shading*). AlphaFold (Abramson et al., 2024) structures of Pah1 (N-LIP, *purple*; HAD-like, *gold*) and the Nem1 (*orange*)-Spo7 (*light blue*) complex are depicted. For simplicity, some domains/regions of the proteins are not shown. *Red* dot represent a phosphate group. (For interpretation of the references to colour in this figure legend, the reader is referred to the Web version of this article.)

Pah1 is a peripheral membrane enzyme that exerts its activity at the nuclear/ER membrane surface (Han et al., 2006; Hosaka and Yamashita, 1984; Karanasios et al., 2010) (Fig. 2). The subcellular location of Pah1 is controlled by the posttranslational modifications of phosphorylation and dephosphorylation (Khondker et al., 2022a) (Fig. 2). In general, the phosphorylated enzyme is localized to the cytosol (Choi et al., 2012; O’Hara et al., 2006); phosphorylation not only serves to sequester Pah1 to the cytosol apart from its membrane-associated substrate PA, but it also protects the enzyme from degradation by the 20 S proteasome (Hsieh et al., 2015; Pascual et al., 2014) (Fig. 2). The recruitment and dephosphorylation of Pah1 at the nuclear/ER membrane is required for its enzyme function (O’Hara et al., 2006; Santos-Rosa et al., 2005; Su et al., 2014b).

### 3.1. Phosphorylation

**Fig. 3.**
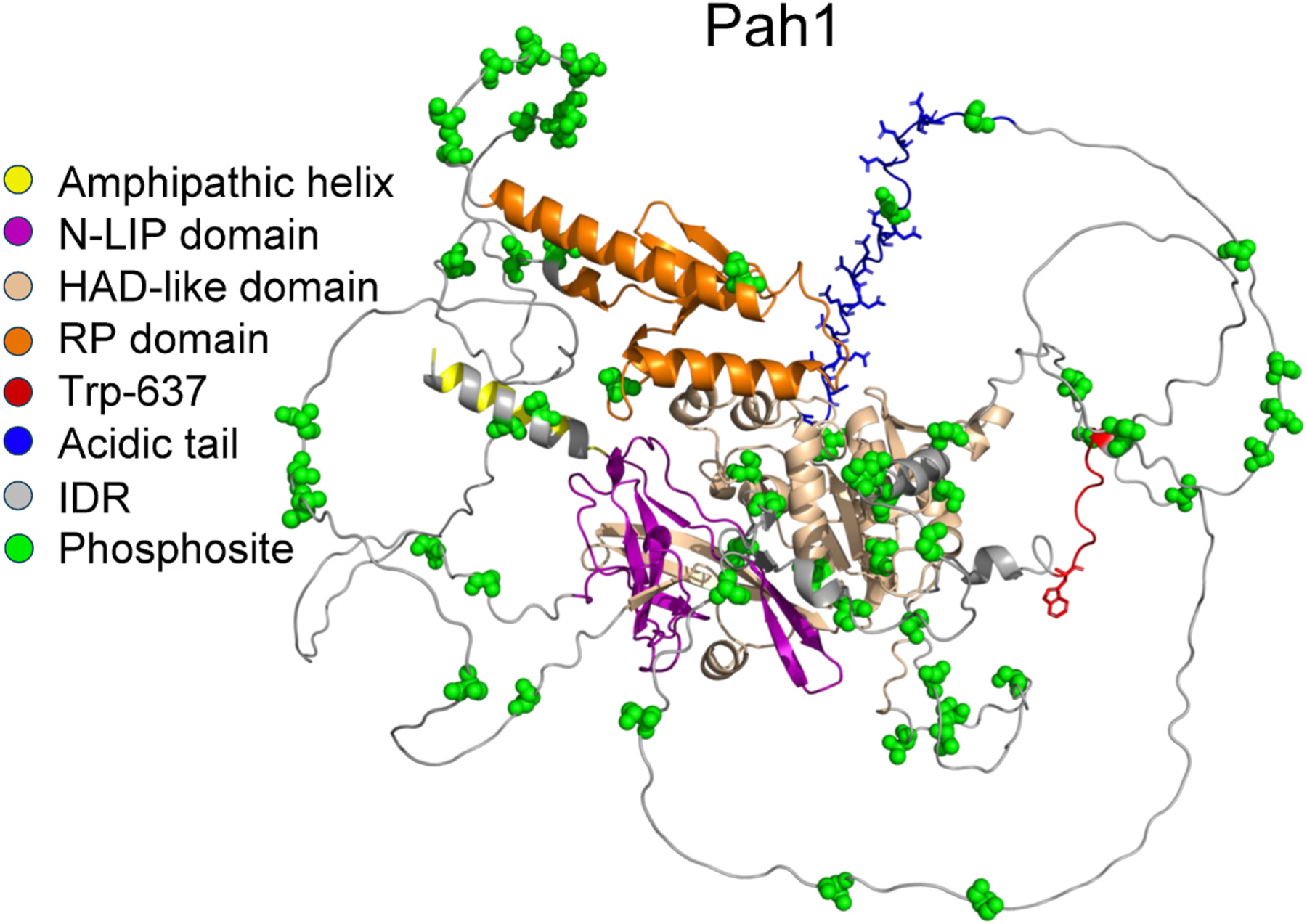
Predicted structure of Pah1. The structure of Pah1 is predicted with AlphaFold and visualized by the PyMol program. The domains, regions, and locations of phosphosites are indicated in the legend.

Pah1 is phosphorylated at 56 serine/threonine residues as mediated by multiple protein kinases (Khondker et al., 2022a). The phosphosites are primarily located within the intrinsically disordered regions (IDRs) and within the HAD-like domain (Fig. 3). The protein kinases that phosphorylate some of these sites have been identified, which include cyclin dependent protein kinases Pho85 (Choi et al., 2012) and Cdc28 (Choi et al., 2011), protein kinases A (Su et al., 2012) and C (Su et al., 2014a), casein kinases I (Hassaninasab et al., 2019) and II (Hsieh et al., 2016), glycogen synthase kinase Rim 11 (Khondker et al., 2022b), and protein kinase Hsl1 (Khondker et al., 2024). Some phosphosites are unique to specific protein kinases while others are common to multiple protein kinases (Khondker et al., 2022a). Some Pah1 phosphorylations are hierarchical in nature, and its phosphorylation at one site affects the phosphorylation at other sites (Khondker et al., 2022a, 2022b). Additionally, phosphorylations by some protein kinases stimulate (e.g., casein kinase I) or inhibit (e.g., Pho85 and Rim11) PAP activity (Choi et al., 2012; Hassaninasab et al., 2019; Khondker et al., 2022b). The understanding of the protein kinase-specific phosphorylation has helped define when (e.g., cell cycle progression, mitotic morphogenesis checkpoint signaling, and meiosis entry) Pah1 is regulated by the posttranslational modification and elucidate the complex regulation for enzyme localization as depicted in Fig. 2 (Khondker et al., 2022a).

### 3.2. Dephosphorylation

The dephosphorylation of Pah1 is catalyzed by the Pah1 phosphatase^2^ that is composed of the Nem1 (catalytic) and Spo7 (regulatory) subunits (O’Hara et al., 2006; Santos-Rosa et al., 2005; Siniossoglou et al., 1998; Su et al., 2014b) (Fig. 2). The Nem1-Spo7 complex at the nuclear/ER membrane activates Pah1 through its recruitment and dephosphorylation (Karanasios et al., 2010; Karanasios et al., 2013; Khondker et al., 2022a; O’Hara et al., 2006; Santos-Rosa et al., 2005; Su et al., 2014b), which permits the enzyme to hop onto the membrane for the recognition and dephosphorylation of PA and to scoot along the membrane for additional rounds of catalysis (Kwiatek and Carman, 2020) (Fig. 2). In addition to its membrane association, dephosphorylated Pah1 is catalytically more active (Su et al., 2014b). Interestingly, Pah1 phosphatase activity is stimulated by the PAP substrate PA (Kwiatek et al., 2022). The protein phosphatase complex shows a higher specificity for the sites of Pah1 phosphorylated by Pho85, but a lower specificity for the sites phosphorylated by protein kinase C (Su et al., 2014b), substantiating PAP regulation by varying signaling pathways.

While the main function of the Pah1 phosphatase is to dephosphorylate Pah1, the protein phosphatase subunits are themselves subject to regulation by phosphorylation (Dey et al., 2019; Su et al., 2018). Both Nem1 and Spo7 are phosphorylated by protein kinases A (Su et al., 2018) and C (Dey et al., 2019). These protein kinases have opposite effects on Nem1-Spo7; its Pah1 phosphatase activity is inhibited by protein kinase A (Su et al., 2018) but stimulated by protein kinase C (Dey et al., 2019). Additionally, target of rapamycin complex 1 (TORC1) indirectly prevents phosphorylation of Nem1 by activating an unknown protein phosphatase or inhibiting an unknown protein kinase that regulates the phosphorylation status of Nem1 (Dubots et al., 2014).

## 4. Structural requirements for Pah1 function and regulation

### 4.1. Pah1

Different domains/regions of Pah1 are associated with its PAP activity, its translocation to, and interaction with the nuclear/ER membrane (Fig. 3). The conserved N-LIP and HAD-like domains comprise the catalytic core required for PAP activity (Han et al., 2006, 2007). The crystal structure of *Tetrahymena thermophila* Pah2, the smallest PAP consisting of only N-LIP and HAD-like domains, shows that the domains co-fold to form a functional catalytic core (Khayyo et al., 2020). The conserved nature of the catalytic core of Pah1 is illustrated by its predicted structure (Fig. 3).

The N-terminal amphipathic helix is responsible for interaction with the membrane, facilitating the active site to recognize PA (Karanasios et al., 2010). The amphipathic helix is presumably hidden when Pah1 is phosphorylated and exposed for membrane association when Pah1 is dephosphorylated (Han et al., 2024; Karanasios et al., 2010). The RP domain facilitates the phosphorylation of the enzyme, which regulates its cellular location and stability (Stukey et al., 2023). A conserved tryptophan (Trp-637) is required for the *in vivo* function (Park et al., 2017, 2022). Trp-637 is involved in the phosphorylation-mediated and dephosphorylation-mediated membrane association of the enzyme (Park et al., 2022). The rest of the Pah1 sequence (i.e., IDRs and acidic tail) is responsible for nuclear/ER localization via interaction with the Pah1 phosphatase (Karanasios et al., 2013; Park et al., 2017; Stukey et al., 2023). The IDRs contain almost all the sites of phosphorylation that govern the interaction with Pah1 phosphatase (Khondker et al., 2022a). The acidic tail, which is rich in negatively charged amino acids, interacts with the Pah1 phosphatase (Karanasios et al., 2013) through ionic interactions with the positively charged amino acids of the Spo7 basic tail (Jog et al., 2024b).

### 4.2. Pah1 phosphatase

**Fig. 4.**
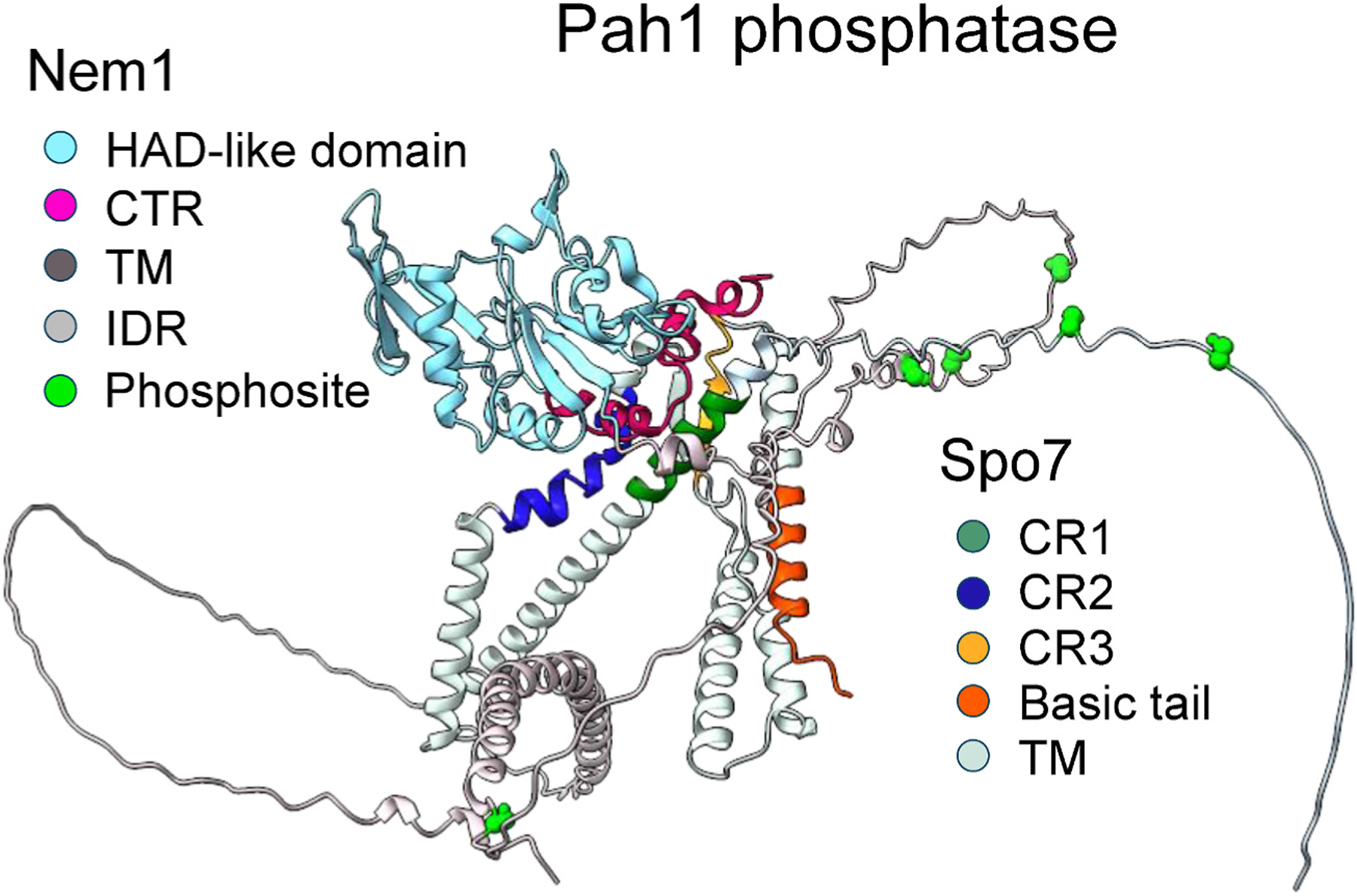
Predicted structure of Pah1 phosphatase. The structure of Pah1 phosphatase, which is composed of Nem1 (catalytic subunit) and Spo7 (regulatory subunit), is predicted with AlphaFold and visualized by the UCSF ChimeraX program. The domains, regions, and locations of phosphosites are indicated in the legends.

The catalytic subunit Nem1 contains the HAD-like catalytic domain, C-terminal region (CTR), and transmembrane regions for membrane association (Jog et al., 2024a; Siniossoglou et al., 1998) (Fig. 4). The CTR contains the conserved hydrophobic residues, which are necessary for the complex formation with the regulatory subunit Spo7. Spo7 contains transmembrane regions for membrane association (Siniossoglou et al., 1998), three conserved regions (CR1, CR2, and CR3), and a basic tail (Jog et al., 2023, 2024b; Mirheydari et al., 2020) (Fig. 4). A hydrophobic sequence in CR1, the hydrophilicity within CR2, and hydrophobicity within CR3 are required for Nem1-Spo7 complex formation (Jog et al., 2023, 2024b; Mirheydari et al., 2020). The C-terminal basic tail containing five arginine and two lysine residues interacts with the acidic tail of Pah1. Mutational effects of the sequences within the Spo7 conserved regions and its basic tail, and Nem1 CTR hydrophobic residues has provided critical information on the importance of the Nem1-Spo7/Pah1 axis in controlling lipid synthesis, lipid droplet formation, nuclear/ER membrane morphology, vacuole fusion, and cell growth (Jog et al., 2023, 2024a, 2024b; Mirheydari et al., 2020).

## 5. PAP as a therapeutic target

### 5.1. Anti-obesity target

The key role that PAP plays in TAG synthesis indicates it may be a potential drug target to ameliorate obesity in humans. Development of PAP inhibitors as an anti-obesity drug is important, but has some limitations. For example, loss of lipin 1 PAP activity results in the loss of body fat and at the same time causes multiple off-target effects that include inflammation, insulin resistance, and peripheral neuropathy (Csaki et al., 2013; Phan and Reue, 2005; Reue and Brindley, 2008; Reue and Donkor, 2007; Reue and Wang, 2019; Suviolahti et al., 2006; Zhang et al., 2014). Thus, it is unclear whether a PAP inhibitor can be identified for use in a systemic manner. Yet, clinically relevant molecules can be delivered to specific cells in drug applications. For example, encapsulation of retinoic acid-generating enzyme aldehyde dehydrogenase into alginate-poly-L-lysine vesicles targets the enzyme to adipocytes, enabling metabolism of vitamin A in the cell (Shen et al., 2018). Interestingly, while lipin PAPs are found in diverse cell types, adipocytes are where lipin 1 is most highly expressed (Donkor et al., 2007). Thus, a similar technology can be developed for adipocyte-specific delivery of the PAP inhibitor to attenuate TAG synthesis potentially without off-target effects.

### 5.2. Antifungal target

Fungal pathogens (e.g., *Cryptococcus neoformans, Candida albicans*, *Candida auris, and Aspergillus fumigatus*) represent a serious threat to human health (Breuer et al., 2022; Fisher et al., 2022; Ivanov et al., 2022; Nelson, 2023; Spivak and Hanson, 2018; Zhai et al., 2012). While some anti-fungal drugs are currently available, the growing frequency of drug-resistant infections clearly requires novel drug development (Breuer et al., 2022; Fisher et al., 2022; Ivanov et al., 2022; Nelson, 2023; Spivak and Hanson, 2018; Zhai et al., 2012). Recent studies have shown that elimination of PAP activity (i.e., *pah1*Δ mutation) is lethal to several fungal pathogens (Jin et al., 2020; Zhao et al., 2022, 2024), indicating that PAP represents a novel target for antifungal drug development. *S. cerevisiae* is commonly used as a model for pathogenic fungi (Bojsen et al., 2012), and importantly, *S. cerevisiae* Pah1 shows strong homology to that of fungal pathogens with respect to the structure of the enzyme and regulation by phosphorylation and dephosphorylation (Jin et al., 2020; Jog et al., 2024b; Mu et al., 2019; Ren et al., 2023; Stukey et al., 2023; Zhao et al., 2024). Thus, information on the inhibitors of *S. cerevisiae* PAP could be applicable to the enzyme of pathogenic fungi.

A consideration in the development of PAP inhibitors as an antifungal drug is that it should be fungal-specific and not affect a host PAP. The acidic tail of Pah1 (Karanasios et al., 2013) and basic tail of Spo7 (Jog et al., 2024b), which are required for the recruitment of Pah1 to its Pah1 phosphatase, and the RP domain, which facilitates the phosphorylation of Pah1 (Stukey et al., 2023) are conserved in opportunistic pathogenic fungi that infect humans, but not conserved in the analogous human enzymes. Inhibitors that target the Pah1 acidic tail (Karanasios et al., 2013) and/or Spo7 basic tail (Jog et al., 2024b) will disrupt PAP function, whereas an inhibitor to the RP domain will result in unregulated detrimental PAP function (Stukey et al., 2023). Thus, such structural feature may be exploited through rational drug design and/or combinatorial chemistry to identify PAP inhibitors that selectively eliminate the pathogens without affecting human PAP activity.

### 5.3. The antidepressant drug sertraline is a novel PAP inhibitor

**Fig. 5.**
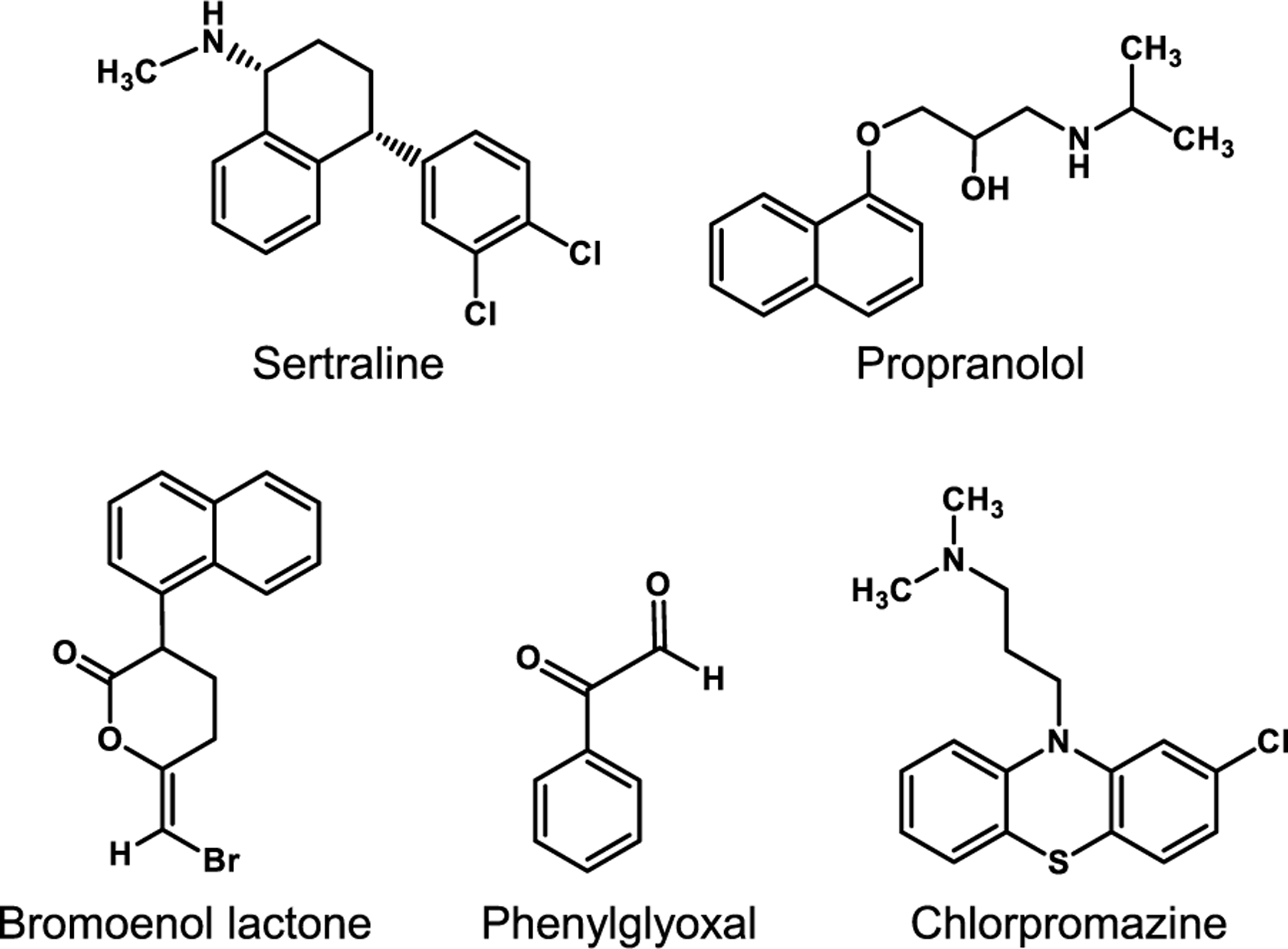
PAP inhibitors. The structures of known PAP inhibitors were obtained from PubChem and redrawn with the ChemDraw program.

Several drugs, which include propranolol, phenylglyoxal, chlorpromazine, and bromoenol lactone, have been used to inhibit PAP activity (Abdel-Latif and Smith, 1984; Eichberg and Hauser, 1974; Fuentes et al., 2003; Grkovich et al., 2006; Jamal et al., 1991; Koul and Hauser, 1987; Morlock et al., 1991) (Fig. 5). Of these, propranolol has received the most attention. For example, propranolol has been useful in showing how PAP influences cellular physiology and disease states in mammalian cells (Asp et al., 2009; Baron and Malhotra, 2002; Brohee et al., 2015; Chae et al., 2020; Dinarvand et al., 2020; Meier et al., 1998) and how the enzyme adversely affects virulence and inhibits growth of pathogenic fungi (Zhao et al., 2024). The antidepressant drug sertraline (McRae and Brady, 2001), which also exhibits broad-spectrum antifungal activity (Zhai et al., 2012), has recently been identified as a novel inhibitor of *S. cerevisiae* Pah1 that rivals the potency of propranolol (Stukey et al., 2024a). In fact, the *K*_*i*_ of sertraline is 7-fold lower than that of propranolol (Stukey et al., 2024a). Consistent with the noncompetitive inhibitory mechanism of sertraline, molecular docking of sertraline predicts that it interacts with non-catalytic residues in the HAD-like domain of Pah1 (Stukey et al., 2024a). Additionally, the activity of the Pah1-CC (catalytic core) variant, which lacks regulatory sequences and independent of Nem1-Spo7-mediated regulation (Han et al., 2024), is inhibited by sertraline in agreement with molecular docking data (Stukey et al., 2024a). That Pah1 is a physiological target of sertraline is supported by the observations that the overexpression of *PAH1* rescues the sertraline-mediated inhibition of *pah1*Δ mutant cell growth, the lethal effect of overexpressing Pah1-CC (Han et al., 2024) is rescued by sertraline supplementation, and that a sublethal dose of the drug results in 2-fold decrease in TAG content (Stukey et al., 2024a). Repurposing existing therapeutics is one option for developing new antifungal strategies (Breuer et al., 2022; Truong et al., 2018; Wall and Lopez-Ribot, 2020), and sertraline appears to be a good candidate for this purpose. Utilizing sertraline in combination therapies with preexisting antifungals may amplify the effects of those drugs prolonging their usefulness as antifungal resistance continues to evolve (Fisher et al., 2012, 2022). Sertraline, however, is not a fungal-specific PAP inhibitor; human lipin 1 (α, β, and γ isoforms) PAP is also inhibited by the drug (Stukey et al., 2024a). This is not surprising given that sertraline targets an allosteric site in the catalytic core of yeast Pah1 (Stukey et al., 2024a) that is conserved in human lipin 1 (Stukey et al., 2024b). Thus, strategies through medicinal chemistry will need to be developed to effect potency and specificity if sertraline is to be used as a PAP inhibitor for fungal and/or human applications.

## Data Availability

All data are contained within the manuscript.

## Footnotes

1 PAP from various organisms are known by different acronyms that are based on the names of genes that encode the enzyme. For *S. cerevisiae*, the protein product of the *PAH1* gene is known as Pah1 (Han et al., 2006), whereas in human and mouse, the protein products of the *LPIN1* and *Lpin1* genes, respectively, are known as lipin 1 (Péterfy et al., 2001). The PAP encoded by *PAH1* differs from the PAP enzymes encoded by *APP1* (Chae et al., 2012; Chae and Carman, 2013), *DPP1* (Toke et al., 1998) and *LPP1* (Toke et al., 1999), which dephosphorylate a broad spectrum of substrates (e.g., phosphatidate, lysophosphatidate, diacylglycerol pyrophosphate, isoprenoid phosphates) and are not involved in *de novo* lipid synthesis (Chae et al., 2012).

2 The analogous Nem1-Spo7 complex in higher eukaryotes is CTDNEP1 (catalytic subunit) and NEP1-R1 (regulatory subunit) (Han et al., 2012; Kim et al., 2007).

## Abbreviations:

PA: phosphatidic acid
PAP: phosphatidic acid phosphatase
DAG: diacylglycerol
TAG: triacylglycerol
HAD: haloacid dehalogenase
IDR: intrinsically disordered region
